# Inadequate Use of Newer Treatments and Glycemic Control by Cardiovascular Risk and Sociodemographic Groups in US Adults with Diabetes in the NIH Precision Medicine Initiative *All of Us* Research Program

**DOI:** 10.1007/s10557-022-07403-2

**Published:** 2022-11-15

**Authors:** Divya Devineni, Meleeka Akbarpour, Yufan Gong, Nathan D. Wong

**Affiliations:** 1 Heart Disease Prevention Program, Division of Cardiology, C240 Medical Sciences, University of California Irvine, Irvine, CA 92697 USA; 2 Department of Epidemiology, University of California, Los Angeles, USA

**Keywords:** Diabetes, SGLT2-inhibitors, GLP-1 receptor agonists, Cardiovascular risk

## Abstract

**Purpose:**

Data are limited on sodium glucose co-transport 2 inhibitors (SGLT2-is) and glucagon-like peptide-1 receptor agonists (GLP-1 RAs) among real-world cohorts of underrepresented patients. We examined these therapies and glycemic control in US adults with diabetes mellitus (DM) by atherosclerotic cardiovascular disease (ASCVD) risk and sociodemographic factors.

**Methods:**

In the NIH Precision Medicine Initiative *All of Us* Research Program, we categorized DM as (1) moderate risk, (2) high risk, and (3) with ASCVD. We examined proportions on DM therapies, including SGLT2-i or GLP-1 RA, and at glycemic control by sociodemographic factors and CVD risk groups.

**Results:**

Our 81,332 adults aged ≥ 18 years with DM across 340 US sites included 22.3% non-Hispanic Black, 17.2% Hispanic, and 1.8% Asian participants; 31.1%, 30.3%, and 38.6% were at moderate risk, high risk, or with ASCVD, respectively. Those with DM and ASCVD were most likely on SGLT2-i (8.6%) or GLP-1 RA (11.9%). SGLT2-i use was < 10% in those with heart failure or chronic kidney disease. The odds (95% CI) of SGLT2-i use were greater among men (1.35 [1.20, 1.53]) and Asian persons (2.31 [1.78, 2.96]), with GLP-1 RA being less common (0.78 [0.70, 0.86]) in men. GLP-1 RA use was greater among those with health insurance, and both GLP-1 RA and SGLT2-i greater within lower income groups. 72.0% of participants had HbA1c < 7%; Hispanic persons were least likely at glycemic control.

**Conclusions:**

Treatment with SGLT2-is and GLP-1 RAs remains low, even among higher ASCVD risk persons with DM and use is even lower among underserved groups.

Cardiovascular diseases (CVD) are the leading causes of death in patients with diabetes mellitus (DM) [1]. Sodium-glucose co-transporter 2 inhibitors (SGLT2-is) and glucagon-like peptide-1 receptor agonists (GLP-1 RAs) are newer DM medications that have shown CVD benefit [2]. SGLT2-is reduce blood glucose by increasing urinary glucose excretion and decreasing blood pressure (BP), weight, and albumin levels [3, 4]. GLP-1 RAs affect glucose control through enhancing glucose-dependent insulin secretion, slowing gastric emptying, and reducing postprandial glucagon and food intake; they decrease weight, C-reactive protein, brain natriuretic peptide, and systolic BP [5–7].

SGLT2-is and GLP-1 RAs are the first DM therapies to show improvement in CVD outcomes from large-scale clinical trials [8]. SGLT2-is reduce overall cardiovascular events [8], heart failure [9], and chronic kidney disease [10]; GLP-1 RAs benefit overall cardiovascular events, but not heart failure [11], with some showing significant reductions in stroke and myocardial infarction [12].

Guidelines have recommended these therapies for reducing the risk of cardiovascular events in those with DM who also have CVD or multiple risk factors [13, 14].

Limited data exist on the extent of use of evidence-based SGLT2-is and GLP-1 RAs among recent real-world cohorts of patients with DM including underrepresented patient groups, and according to level of cardiovascular risk and sociodemographic factors. In this study, we examine the use of these therapies and glycemic control among a large and diverse contemporary cohort of US adults with DM.

## Methods

### All of Us Research Program

The *All of Us* Research Program is part of the National Institutes of Health Precision Medicine Initiative with planned enrollment of one million participants throughout the USA and emphasis in recruiting those from disadvantaged backgrounds. The mission of the *All of Us* Research Program is to accelerate health research and medical breakthroughs, enabling individualized prevention, treatment, and care [15]. There are currently over 417,000 participants that have been recruited from 370 + sites nationwide through 2022, with enrollment starting in 2018. Over 50% of these participants represent racial and ethnic minorities, and over 80% of them are underrepresented in biomedical research [15]. Participants were recruited through convenient registration and enrolled digitally through the *All of Us* website [16]. They provided consent to access of EHR (electronic health record) data and completed surveys for demographic and health data [16]. The *All of Us* Research Program was approved by the institutional review boards of all participating sites and informed consent was provided by all participants. The current analysis utilized de-identified data approved for use by researchers at participating sites.

We utilized data collected using the *All of Us* Researcher Workbench, a cloud-based platform for use by approved researchers [17]. The data includes surveys, EHR data, and physical measurements (PM). The EHR data included medical conditions, drug exposures, procedures, and labs/measurements. The survey data includes demographic information, lifestyle questions, personal medical history, healthcare access and utilization, family health history, and overall health. Participants could choose not to answer specific questions. PM recorded at enrollment include systolic and diastolic blood pressure, height, weight, heart rate, waist and hip measurement, wheelchair use, and current pregnancy status [17]. The program includes participants of different ethnicity/race, age, and geographic areas in the USA, as well as different education, income, gender identity, and health status [17]. The *All of Us* Research Program emphasizes the importance of social determinants of health and addressing the health of underserved populations. Since these populations have historically been excluded from research, this program aims to understand how to properly treat and prevent diseases in people of all backgrounds [15].

### Study Sample

We identified a cohort of 81,332 participants aged ≥ 18 years enrolled during 2018–2022 with DM defined based on ≥ 1 of the following: insulin treatment or diabetes medication (*n* = 65,584), medical or personal history (*n* = 49,408), and/or laboratory values (HbA1c ≥ 6.5%, fasting glucose ≥ 126 mg/dL, or non-fasting glucose ≥ 200 mg/dL) (*n* = 30,161). We excluded participants with type 1 DM and variables with missing values in our analysis from participants. Race/ethnicity within our cohort included non-Hispanic White, non-Hispanic Black, Hispanic or Latino, and Asian. We categorized our CVD risk groups as moderate risk based on ≤ 1 CVD risk factor, high risk with ≥ 2 CVD risk factors, and DM with known CVD. Risk factors included were age ≥ 60 years, hypertension (blood pressure ≥ 130/80 mmHg or being on antihypertensive therapy), low-density lipoprotein cholesterol (LDL-C) ≥ 160 mg/dL, cigarette smoking, and high-density lipoprotein cholesterol (HDL-C) < 40 mg/dL for males and < 50 mg/dL for females. Across CVD risk groups, ethnicity, and sex, we examined the percent on any and specific DM therapies, on SGLT2-i and GLP-1 RA, and at different levels of hemoglobin A1c (HbA1c). We also analyzed these parameters across health insurance status, education, and income.

### Definition and Measurements

We included information on demographics, body mass index (BMI), blood pressure, triglycerides, LDL-C, HDL-C, smoking, glomerular filtration rate (GFR), hemoglobin A1c (HbA1c) and glucose, heart failure (HF), atherosclerotic CVD (ASCVD), DM medication, survey data, and cholesterol. ASCVD was defined as a history of coronary artery disease, cerebrovascular disease (excluding hemorrhagic stroke), and peripheral arterial disease. DM medication included insulins and analogues and other oral diabetes medication. GLP-1 RA therapies included were albiglutide, dulaglutide, exenatide, liraglutide, lixisenatide, and semaglutide. SGLT2-i medications included canagliflozin, dapagliflozin, empagliflozin, and ertugliflozin. Medication use was collected through EHRs and surveys, which included medication use at any point. We also obtained survey data on health insurance status, types of health insurance, cigarette smoking status, and income.

### Statistical Analysis

We utilized the R statistical package provided within the research workbench for the *All of Us* Research Program. The chi-squared test of proportions was used to compare use of DM therapies and HbA1c control by risk groups, sex, race/ethnicity, as well as by health insurance, education, income categories, and year of study enrollment based on survey. We categorized continuous variables such as HbA1c into clinically relevant groups. Across the different groups, we examined the percent on any DM therapy, on SGLT2-i and GLP-1 RA, and with HbA1c < 7%, 7 to < 8%, and ≥ to 8%. We also studied the percent on SGLT2-i among those with heart failure (HF) or chronic kidney disease (CKD) (eGFR < 60 mL/min/1.73 m^2^). Multiple logistic regressions were used to assess the relation of predetermined sociodemographic factors, risk groups, and individual risk factors with the use of newer DM therapies, with odds ratios (ORs) and 95% confidence intervals calculated.

## Results

We included 81,332 participants diagnosed with DM based on our inclusion criteria. Overall, 31.1%, 30.3%, and 38.6% were at moderate risk, high risk, or with ASCVD, respectively. Our sample also comprised 22.3% non-Hispanic Black, 17.2% Hispanic or Latino, and 52.3% non-Hispanic White, 1.8% Asian participants, as well as 40.6% males and 59.4% females. Overall, 4.4% did not have health insurance and 34.1% had a high school or less education (Table 1).

**Table 1:** Demographic characteristics of participants with type 2 DM

Variable	Total (*N* = 81,332)
Age (years) (mean +/- SD)	62.0 (± 14.1)
Male	31,887 (40.6%)
Female	46,661 (59.4%)
Non-Hispanic White	42,532 (52.3%)
Non-Hispanic Black	18,100 (22.3%)
Hispanic or Latino	13,986 (17.2%)
Asian	1445 (1.8%)
Other race/ethnicity	5269 (6.5%)
Has health insurance	74,838 (95.6%)
Income	
Less than 10 k	11,678 (19.6%)
10 k–25 k	11,793 (19.8%)
25 k–35 k	5994 (10.1%)
35 k–50 k	6262 (10.5%)
50 k–75 k	7990 (13.4%)
75 k–100 k	5673 (9.5%)
More than 100 k	10,046 (16.9%)
Education	
Less than a high school degree or equivalent	9527 (12.2%)
Twelve or GED	17,147 (21.9%)
Some college	22,394 (28.6%)
College graduate/advanced degree	29,104 (37.2%)
BMI (kg/m^2^) (mean +/- SD)	32.5 (± 12.2)
Smoking status	
Non-smoker	43,071 (54.7%)
Former smoker	23,383 (29.7%)
Current smoker	12,229 (15.5%)
Systolic blood pressure (mm Hg) (mean +/- SD)	129.5 (± 14.2)
Diastolic blood pressure (mm Hg) (mean +/- SD)	76.9 (± 9.1)
Triglycerides (mg/dL) (mean +/- SD)	145.7 (± 85.1)
LDL-C (mg/dL) (mean +/- SD)	100.9 (± 31.3)
HDL-C (mg/dL) (mean +/- SD)	50.1 (± 15.2)
Heart failure	12,826 (15.8%)
Systolic heart failure	5141 (6.3%)
Diastolic heart failure	6863 (8.4%)
Estimated glomerular filtration rate (eGFR) (mean +/- SD)	83.3 (± 23.3)
Diabetes risk and ASCVD status	
≤ 1 diabetes risk factors without ASCVD	24,787 (31.1%)
≥ 2 diabetes risk factors without ASCVD	24,112 (30.3%)
Diabetes with ASCVD	30,682 (38.6%)
Microvascular complications	
Retinopathy	4991 (6.1%)
Neuropathy	9912 (12.2%)
Nephropathy	10,660 (13.1%)
Diabetes risk factors	
Age ≥ 60 years	50,768 (62.4%)
Has HTN	42,315 (54.9%)
LDL-C ≥ 160 mg/dL	1835 (3.4%)
Smoking history	12,229 (15.5%)
HDL-C < 50 mg/dL in females	14,861 (18.3%)
HDL-C < 40 mg/dL in males	8641 (10.6%)

Within ASCVD risk groups, sex, and ethnicity, there were significant differences (*p* < 0.001) in the use of any DM medication use, SGLT2-i and GLP-1 RA use, as well as in use of metformin and insulin (Table 2). Non-Hispanic Black and Hispanic or Latino people had the highest proportion on any DM medication (*p* < 0.001), and there was a higher percentage among non-Hispanic Black and Hispanic or Latino people compared to non-Hispanic White people on SGLT2-is or GLP-1 RAs. Other race/ethnicity participants had the highest proportion on GLP-1 RA with 11.4%, and Asian participants had the highest proportion on SGLT2-i with 9.7%. Those with DM and ASCVD had the highest proportion (*p* < 0.001) of participants using SGLT2-i (8.6%) and GLP-1 RA (11.9%) analyzed. There was a higher proportion of females (*p* < 0.001) on GLP-1 RAs (11.3%); however, there was a higher proportion of males on SGLT2-is (7.3%.). Metformin use had the highest proportion (*p* < 0.001) among females (37.6%), those at moderate risk (39.6%), and non-Hispanic Black people (43.6%). Insulin use had the highest proportions among males (41.7%), those with DM and ASCVD (50.4%), and non-Hispanic Black people (48.7%) (*p* < 0.001 across sex, race/ethnicity, and ASCVD groups). There were also significant differences (*p* < 0.001) across all groups for patients with heart failure on SGLT2-is, with the highest percentages in those with DM who had ASCVD (8.0%), males (8.3%), and Asian persons (14.4%).

**Table 2:** Prevalence of diabetes treatments and glycemic control in adults with diabetes across risk groups, sex, and ethnicity

Proportion (%)	Total (*N* = 81,332)	≤ 1 diabetes RF w/o ASCVD (*N* = 24,787)	≥ 2 diabetes RF w/o ASCVD (*N* = 24,112)	Diabetes with ASCVD (*N* = 30,682)	Female (*N* = 46,661)	Male (*N* = 31,887)	Non-Hispanic White (*N* = 42,532)	Non-Hispanic Black (*N* = 18,100)	Hispanic or Latino (*N* = 13,986)	Asian (*N* = 1445)	Other race/ethnicity (*N* = 5269)
Any DM medication	63.3%	60.9%	60.6%	67.5%*	64.0%	62.3%*	57.8%	72.5%	67.8%	64.6%	64.1%*
SGLT2-i or GLP-1 RA	13.5%	10.7%	12.9%	16.3%*	13.9%	12.9%*	12.5%	14.6%	14.5%	15.1%	14.7%*
SGLT2-i and GLP-1 RA	3.1%	2.0%	2.8%	4.2%*	3.0%	3.2%	2.9%	3.3%	3.3%	4.2%	3.6%*
GLP-1 RA	10.3%	8.7%	10.0%	11.9%*	11.3%	8.9%*	9.7%	11.3%	10.6%	9.6%	11.4%*
SGLT2-i	6.3%	4.0%	5.8%	8.6%*	5.7%	7.3%	5.7%	6.6%	7.2%	9.7%	6.9%*
Insulin use	38.9%	31.6%	32.2%	50.4%*	37.0%	41.7%*	33.7%	48.7%	42.7%	30.6%	39.0%*
Metformin	36.2%	32.3%	39.6%	36.6%*	37.6%	34.1%*	30.7%	43.6%	42.4%	43.0%	35.9%*
Dipeptidyl peptidase 4 (DPP4)	6.8%	4.2%	7.0%	9.0%*	7.0%	6.7%	5.4%	8.4%	8.7%	8.8%	6.9%*
HbA1c categories											
< 7%	72.0%	77.6%	71.5%	68.9%*	73.6%	69.8%*	78.3%	65.1%	60.8%	76.3%	73.1%*
7–8%	11.9%	8.7%	12.0%	13.9%*	11.0%	13.3%*	11.4%	11.9%	13.5%	14.3%	11.4%*
≥ 8%	16.1%	13.7%	16.5%	17.2%*	15.4%	17.0%*	10.3%	22.9%	25.7%	9.3%	15.5%*
Pts w/ heart failure (*n* = 11,501) on SGLT2-i	7.3%	4.5%	5.3%	8.0%*	6.4%	8.3%*	7.0%	6.3%	9.7%	14.4%	6.8%*
Pts w/CKD w/eGFR < 60 (*n* = 2057) on SGLT2-i	9.9%	4.7%	7.6%	11.6%	8.8%	11.5%^†^	10.2%	9.7%	7.4%	0.0%	11.6%

^†^*p* value < 0.05, *p < .001 across risk, sex, or ethnic groups. Participants may be on one or more medication class

Table 3 shows DM medication use according to health insurance, education, and income status. Those who have health insurance had a higher proportion on both SGLT2-i (6.4%) (*p* < 0.001) and GLP-1 RA (10.5%) (*p* < 0.001). By education, those who went to college had the highest percentage on GLP-1 RA at 11.6% (*p* < 0.001), but those who had less than a high school degree had the highest percentage on SGLT2-i at 7.1% (*p* < 0.001). For the income groups, those who are earning 10 k–25 k annually had the highest proportion on GLP-1 RA at 12.1% (*p* < 0.001) and on SGLT2-i at 7.3% (*p* < 0.001). Since the GLP-1 RAs liraglutide and semaglutide have been approved for use in obesity, we analyzed use of GLP-1 RA and SGLT2-i by BMI status (not shown in table). Those with a BMI of ≥ 30 kg/m^2^, classified as obese, had the highest proportion on GLP-1 RA (15.2%) (*p* < 0.001) and on SGLT2-i (8.1%) (*p* < 0.001) compared to other BMI classes. The proportion on each respectively showed an increasing trend with each BMI class from underweight to obese. In addition, Table 4 compares use of newer and older glucose-lowering therapies as well as HbA1c categories across enrollment year (based on year of survey completion). While most comparisons showed statistically significant differences across study years, the absolute differences were quite modest with no demonstrable trend of increasing or decreasing use of any of the therapies or glycemic control across study years.

**Table 3:** Prevalence of diabetes treatments and glycemic control in adults with diabetes across health insurance, education, and income

Proportion (%)	Any DM medication use	SGLT2-i or GLP-1 RA use	SGLT2-i and GLP-1 RA use	GLP-1 RA use	SGLT2-i use	Metformin use	Insulin use	DPP4 use
Health insurance (*N* = 7438)	63.1%	13.7%	3.2%	10.5%	6.4%	35.9%	38.6%	6.9%
No health insurance (*N* = 3469)	68.4%*	9.7%*	2.0%*	6.9%*	4.8%*	45.0%*	43.5%*	6.2%
Less than a high school degree (*N* = 9527)	71.5%	13.1%	2.8%	8.8%	7.1%	44.8%	49.1%	9.5%
Twelfth grade or GED (*N* = 17,147)	66.5%	13.2%	3.1%	10.0%	6.3%	38.2%	44.2%	7.2%
College (*N* = 22,394)	64.7%	14.7%	3.4%	11.6%	6.5%	36.8%	41.2%	7.1%
College graduate/advanced degree (*N* = 29,104)	57.4%*	12.9%*	3.0%^†^	10.0%*	5.9%*	31.5%*	30.4%*	5.6%*
Income less than 10 k (*N* = 11,678)	71.6%	13.1%	3.0%	10.0%	6.1%	41.5%	48.6%	7.9%
10 k–25 k (*N* = 11,793)	68.7%	15.8%	3.6%	12.1%	7.3%	40.0%	45.6%	8.8%
25 k–35 k (*N* = 5994)	65.2%	14.5%	3.1%	11.0%	6.6%	38.6%	40.9%	7.3%
35 k–50 k (*N* = 6262)	60.8%	14.5%	3.3%	11.6%	6.2%	36.8%	35.8%	6.6%
50 k–75 k (*N* = 7990)	60.0%	14.1%	3.3%	10.7%	6.7%	35.3%	33.5%	5.7%
75 k–100 k (*N* = 5673)	57.4%	13.5%	3.5%	10.6%	6.3%	31.7%	30.2%	5.7%
More than 100 k (*N* = 10,046)	54.1%*	12.4%*	3.0%	9.7%*	5.7%*	28.1%*	25.8%*	4.7%*

^†^*p* value < 0.05, *p < .001 across health insurance, education, or income. Participants may be on one or more medication class

**Table 4:** Prevalence of diabetes treatments and glycemic control in adults with diabetes across years of enrollment (based on survey completion year)

Proportion (%)	Total (*N* = 81,332)	2018^1^ (*N* = 43,568)	2019 (*N* = 26,142)	2020 (*N* = 6336)	2021 (*N* = 5291)
Any DM medication	63.3%	63.6%	63.9%	61.8%	59.9%*
SGLT2-i or GLP-1 RA	13.5%	13.5%	13.4%	14.3%	13.5%^†^
SGLT2-i and GLP-1 RA	3.1%	3.1%	3.2%	2.9%	3.1%
GLP-1 RA	10.3%	10.3%	10.2%	10.9%	10.4%^†^
SGLT2-i	6.3%	6.3%	6.4%	6.3%	6.2%^†^
Insulin use	38.9%	39.6%	38.8%	36.8%	36.0%*
Metformin	36.2%	36.5%	36.7%	34.1%	32.8%*
Dipeptidyl peptidase 4 (DPP4)	6.8%	7.0%	6.9%	6.0%	5.6%*
HbA1c categories					
< 7%	72.0%	71.5%	73.0%	71.7%	71.3%*
7–8%	11.9%	11.9%	11.7%	13.3%	12.3%
≥ 8%	16.1%	16.6%	15.4%	15.0%	16.4%

^1^Also includes patients with completed survey information from earlier years

^†^*p* value < 0.05, *p < .001 across groups

**Fig. 1:**
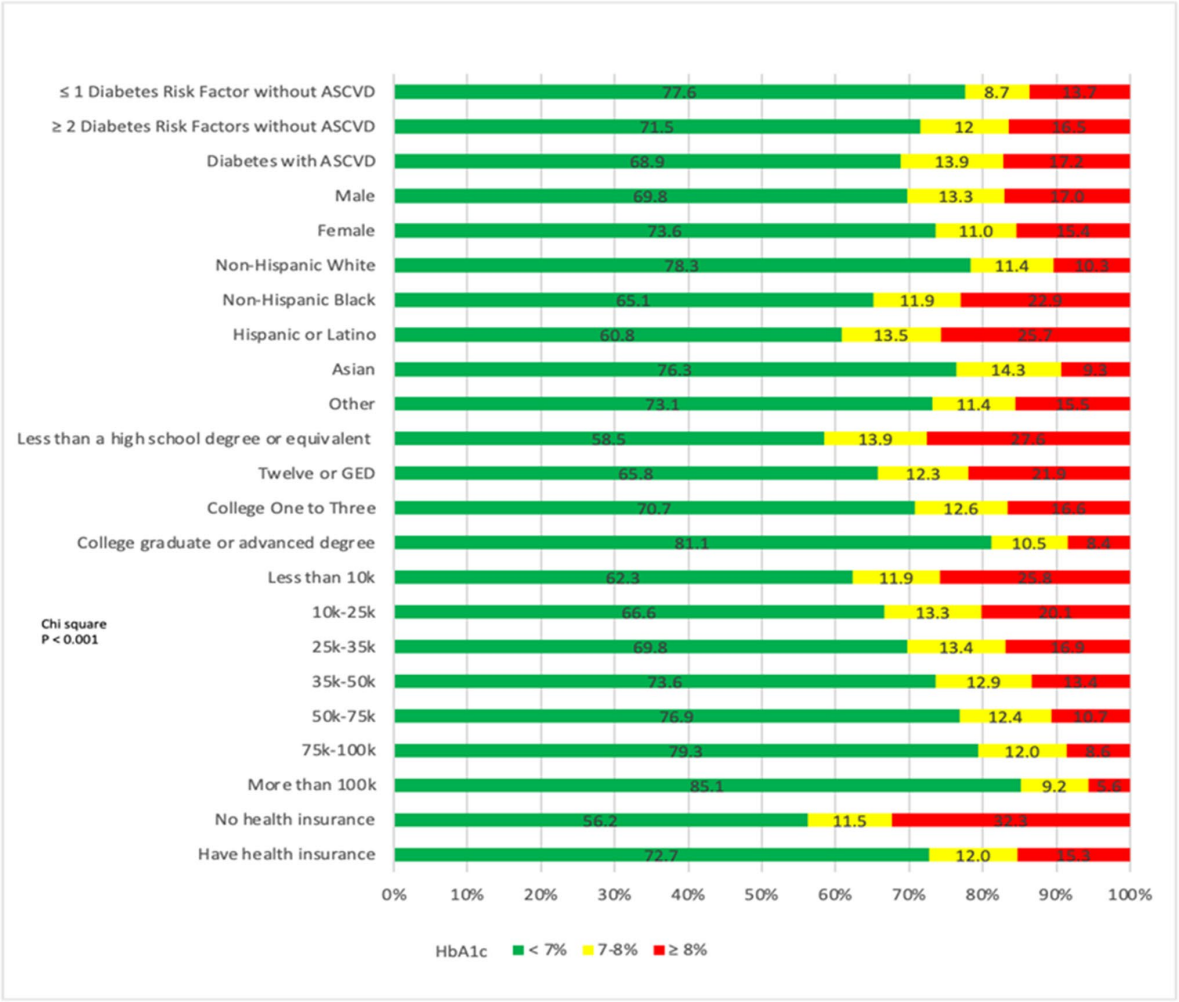
Proportion of participants at ideal, borderline, or poor HbA1c control by ASCVD risk group, sex, ethnicity, education, income, and health insurance results. *p* < 0.001 across risk, ethnicity, sex, education, income, and health insurance status categories

Figure 1 shows proportions of participants according to HbA1c level (< 7%, 7– < 8%, and ≥ 8%) stratified by ASCVD risk group, sex, ethnicity, income level, education, and insurance status. Approximately 17% of people with DM with ASCVD in the *All of Us* participants have HbA1c ≥ 8%. Furthermore, another 14% were at modest, but not ideal control with HbA1c between 7 to less than 8%. Over a fourth of (25.7%) Hispanic or Latino participants and 17.0% of males were not at ideal HbA1c control, defined as greater than or equal to 8%. Those with less than a high school degree or equivalent had the highest proportion of glucose not at control at 27.6%. Participants with no health insurance had the higher percentage not controlled at 32.3%, and the lowest income bracket of less than 10 k had a highest percentage not controlled at 25.8% (*p* < 0.001 across ASCVD risk, ethnicity, sex, education, income, and health insurance status categories).

From multiple logistic regression analyses (Table 5), higher income participants of 75 k - 100 k were more likely to be on GLP-1 RA (OR = 1.28 [1.11, 1.47]), but lower income participants of 10 k - 25 k were likely to be on SGLT2-i (OR = 1.20 [1.05, 1.36]), with a reference group of income < 10 k. In addition, the DM risk group with ASCVD were more likely to be on SGLT2-is (OR = 1.64 [1.43, 1.88]) and GLP-1 RAs (OR = 1.21 [1.09, 1.34]), with a reference group of < 1 DM risk factor. Those who have health insurance were more likely to be on GLP-1 RA (OR = 1.40 [1.16, 1.70]), but not on SGLT2-i (OR = 0.98 [0.79, 1.23]), with participants having no health insurance as the reference group. For ethnicity, Asian participants were more likely to be on SGLT2-i (OR = 2.31 [1.78, 2.96]), but other participants were more likely to be on GLP-1 RA (OR = 1.25 [1.07, 1.46]), with a reference group of non-Hispanic White participants. Lastly, male participants (OR = 1.35 [1.20, 1.53]) were more likely to be on SGLT2-i, but were less likely to be on GLP-1 RA (OR = 0.78 [0.70, 0.86]), with a reference group of female participants.

**Table 5:** Multiple logistic regression of indicators for SGLT2-i and GLP-1 RA use

Variable	SGLT2-i odds ratio [95% CI]	GLP-1 RA odds ratio [95% CI]
Age (per year)	1.00 [0.995, 1.07]	0.99 [0.98, 0.99]
Gender: male	1.35 [1.20, 1.53]	0.78 [0.70, 0.86]
BMI (per kg/m^2^)	1.006 [1.004, 1.009]	1.03 [1.03, 1.03]
Age ≥ 60 years	0.93 [0.80, 1.08]	0.91 [0.81, 1.02]
HTN	1.04 [0.95, 1.15]	1.15 [1.07, 1.24]
LDL-C ≥ 160 mg/dL	0.63 [0.47, 0.82]	0.70 [0.57, 0.86]
Smoking history	0.67 [0.58, 0.76]	0.78 [0.71, 0.87]
HDL-C < 50 mg/dL in females	1.93 [1.71, 2.17]	1.64 [1.50, 1.79]
HDL-C < 40 mg/dL in males	1.69 [1.48, 1.92]	1.90 [1.69, 2.13]
Ethnicity: non-Hispanic Black	1.43 [1.28, 1.60]	1.12 [1.03, 1.22]
Hispanic or Latino	1.67 [1.47, 1.89]	1.16 [1.05, 1.29]
Asian	2.31 [1.78, 2.96]	0.93 [0.70, 1.21]
Other	1.43 [1.18, 1.73]	1.25 [1.07, 1.46]
Have health insurance	0.98 [0.79, 1.23]	1.40 [1.16, 1.70]
Income: 10 k–25 k	1.20 [1.05, 1.36]	1.22 [1.10, 1.36]
25 k–35 k	1.08 [0.91, 1.27]	1.24 [1.09, 1.41]
35 k–50 k	0.99 [0.84, 1.17]	1.26 [1.11, 1.43]
50 k–75 k	1.15 [0.98, 1.34]	1.18 [1.04, 1.33]
75 k–100 k	1.14 [0.96, 1.36]	1.28 [1.11, 1.47]
More than 100 k	0.99 [0.84, 1.16]	1.22 [1.07, 1.38]
DM risk group: ≥ 2 diabetes risk factors w/o ASCVD	1.10 [0.95, 1.28]	0.95 [0.84, 1.06]
Diabetes with ASCVD	1.64 [1.43, 1.88]	1.21 [1.09, 1.34]

**Reference groups:** Gender—female, age ≥ 60 years—age ≤ 60 years age, HTN—no HTN, LDL-C ≥ 160 mg/dL—LDL-C ≤ 160 mg/dL, smoking history—no smoking history, HDL-C < 50 mg/dL in females—HDL-C > 50 mg/dL in females, HDL-C < 40 mg/dL in males—HDL-C > 40 mg/dL in males, race—non-Hispanic White, health insurance—none, income— < 10 k, DM risk group— ≤ 1 diabetes risk factor

## Discussion

Our study in a large real-world cohort of US adults of diverse backgrounds shows that across all ASCVD risk groups, sexes, and ethnic groups, there remains significant underutilization of SGLT2-is and GLP-1 RAs. While use of these therapies was higher in those with DM who had multiple risk factors or ASCVD compared to those without multiple risk factors, even in those with both DM and ASCVD, only 8.6% were on an SGLT2-i and 11.9% on a GLP-1 RA. Although the use of SGLT2-i or GLP-1 RA was slightly greater in Hispanic or Latino people than in non-Hispanic White persons, barely one-tenth of all ethnic groups with DM are on these newer therapies. Those without health insurance or with lower levels of educational attainment were also least likely to be on these therapies.

A recent study focusing on persons with health insurance found similar results to our study with low use of SGLT2-i and GLP1-RA among patients with DM [19]. Similarly, among those with DM in the National Health and Nutrition Examination Survey 2017–2018, only 4.5% were on SGLT2-i and 1.5% were on GLP-1 RA [20]. Specifically, in those with ASCVD and type 2 DM, others found < 12% to be on these newer DM medications [21]. The results from our study as well as others are concerning as CVD outcomes are higher among those on sulfonylureas or insulin compared to those on GLP-1 RAs, SGLT2-is, and DPP-4 inhibitors [22].

Data from the Diabetes Collaborative Registry (2013–2016) showed that metformin, sulfonylureas, and insulin were used more by patients with type 2 diabetes compared to SGLT2-is and GLP-1 RAs, which were used by only 4.2% and 5.5% respectively by DM patients in the registry [23]. Among these patients without heart failure, only 4.2% and 5.5% were on SGLT2-i and GLP-1 RA, respectively [23]. Furthermore, only 2.7% and 4.3% were on SGLT2-i and GLP-1 RA, respectively among patients with both type 2 DM and heart failure [23], showing clear underutilization of these newer therapies. While there are some slight increases in the proportion of type 2 DM patients with CVD taking SGLT2-i and GLP-1 RA since the publications of EMPA-REG OUTCOME and LEADER, use of these medications is still quite limited [24]. From 2015 to 2018, patients with type 2 DM and CVD from the Optum Claims Database show an increase from 4.1 to 7.2% on SGLT2-i and 4.2 to 8.2% on GLP-1 RA [24].

Clinical inertia remains an important reason for the underutilization of these therapies. This includes cardiologists’ skepticism about prescribing diabetes medications; some feel these are out of their scope of practice [18]. Furthermore, without proper knowledge on these therapies, there is hesitancy about prescribing them [18]. Finally, there is a lack of implementation of automated systems approaches, such as electronic medical record reminders and best practice advisories that can be helpful to ensure their appropriate utilization.

Our data underscore the need to better disseminate and implement recent guidelines that focus on the use of these therapies for those with DM at highest risk of CVD [2]. While the highest use of therapies was in those with DM and ASCVD, use was still seriously inadequate with < 15% of such persons on these therapies. There is often a lag of more than 5 years between recommendation of newer medications and even modest adoption of their use [25]. The guidelines to use the newer medications are only a few years old, so our results are not surprising. The value of SGLT2-i therapies in reducing CVD outcomes has been reported with empagliflozin and canagliflozin among those with both DM at high CVD risk [26, 27], and dapagliflozin in mostly primary prevention DM for reducing cardiovascular death or hospitalization due to heart failure [9]. Similarly, the value of GLP-1 RA therapies in reducing adverse outcomes has been shown with liraglutide in those with DM with and without CVD [28], as well as subcutaneously administered semaglutide [12], but not oral semaglutide [29]. However, not all SGLT2-is and GLP-1 RAs have research and trial data to back their use in reducing occurrence of ASCVD.

While SGLT2-i use was somewhat higher in our participants with HF who were males, Hispanic or Latino persons, use was still suboptimal with use at only 6–10% for most groups. Those with CKD had even less usage of SGLT2-is, with no significant difference in usage across all different categories. This could be explained by in part by older persons being less likely to initiate SGLT2-i or GLP-1 RA treatment, despite the high prevalence of CKD in this population [30]. Older patients are more likely to have other health contraindications for starting these therapies and could be skeptical of newer treatments. Since the greatest concentration of those with CKD and diabetes are in this population, the under use of SGLT2-i in our data could be related to this discrepancy.

We identified HbA1c control remains suboptimal, especially among those with DM and CVD who are a highest risk. Since around 70% of our participants were controlled according to HbA1c, it was not surprising that the use of SGLT2-i and GLP-1 RA was low. Hispanic or Latino persons as well as Asian persons had the highest proportions not in glycemic control. Previous research has shown that both Hispanic or Latino persons and Asian persons have higher HbA1c levels compared to White persons [31], and there are multiple explanations for the continued elevated HbA1c levels among these ethnic groups. One explanation is that there is a lack of culturally tailored diabetes prevention program for specific ethnic groups, which needs to address ethnic-specific dietary and physical habits, as well as risk factors that are most predominant among specific groups [32]. Similarly, a systemic lack of diabetes support resources, guidance, and mental health well-being plays a role in the poor glycemic control in the Hispanic or Latino population [33]. Not surprisingly, we noted those of higher income to be more likely on GLP-1 RAs, consistent with the notion that those who can afford newer therapies will be on them [34]. Furthermore, given the extreme expense of these medications, our results are not too surprising [35]. These agents are often held in reserve for later use when traditional medications are ineffective. We also show that the highest risk group of diabetes with ASCVD are also more likely to be on both therapies, following the trend expected after the recent guidelines on medications for at high-risk groups [2]. Finally, our observations showing a lack of greater uptake of these therapies nor improved glycemic control over time underscores the continued disparities in diabetes treatment among our cohort with a high representation of underserved, lower education, and lower income participants.

We show that those on health insurance are more likely to be on both SGLT2-i and GLP-1 RA. Research has shown that patients with commercial health insurance plans are more likely to be on these newer diabetes therapy, specifically SGLT2-is [36]. Similarly, those with private health insurance plans are more likely initiate treatment on GLP-1 RA [37]. However, the mere presence of health insurance may be insufficient; Medicare Advantage health insurance plans may only partly cover the costs of these medications, resulting in significant out-of-pocket costs [38]. Furthermore, patients without health insurance are least likely to have access to these therapies. Financial barriers to obtaining many beneficial medications remain an important cause of adverse health outcomes globally.

Interestingly, in adjusted analyses, we found that Asian, Black, and Hispanic/Latino participants have greater odds of being on SGLT2-is and Black and Hispanic/Latino participants have greater odds of being on GLP1-RAs than White participants, although use in all groups remained low. Further studies on ethnic/racial medication preferences as well as responses to new therapies are needed. Similarly, we noted that males were more likely to be on SGLT2-i, but not on GLP-1 RA. Females were not prescribed SGLT2-is as often as males, suggesting that gender inequality influences therapeutic plans, which ultimately results in detrimental outcomes and is reflected in our results [34]. Additionally, the mechanism of action of SGLT2-is involves increases in renal glucosuria, which creates a favorable environment for pathogens to proliferate, leading to genital infections. There has been a higher incidence of genital infections after initiating SGLT2-is in female patients [39], which could explain the sex differences in SGLT2-i use in our study.

Inequalities in use of these medications also have to do with underserved populations not having adequate access to these therapies. Special programs are needed by payers and manufacturers of these drugs to make these more available to underserved populations with low utilization. Since a major challenge is getting these newer therapies to underserved populations, some of the ways this can be accomplished is through studying the social determinants of health in individual communities, incorporating virtual care, and shifting budgets to outpatient/primary-care settings [40]. Furthermore, focusing on value-based care and evidence-based medicine, along with a single-payer model and a public choice model, can be utilized to reach universal coverage [41].

Our study has several strengths and limitations. Our study is unique in that the participants reflect the diversity of the USA, including significant representation of people who have not taken part in or have been left out of health research before, including persons representative of the diversity of the USA from all backgrounds and regions. The detailed linkage to medical records allows assessment of SGLT2-i and GLP-1 RA use and glycemic control across groups stratified by ASCVD risk as well as social determinants of health. A limitation is the cross-sectional nature of our study without multiple measures to assess adherence, nor follow-up to examine cardiovascular or mortality outcomes. Furthermore, since our sample size is large, small differences reach the threshold of statistical significance.

In summary, our study has shown in a diverse cohort of US adults that despite recent guidelines for SGLT2-is or GLP-1 RAs use in higher risk people with DM, few are actually taking them, and use is lower among the underserved. Several large clinical trials over the last decade have demonstrated greater benefit in using new glucose lowering therapies than those previously recommended for standard care. We would expect these results to lead to increased uptake over time of said newer treatments, but data relating to use of these agents in a real-world setting are limited. Our study emphasizes the need to further examine barriers towards implementation of these newer therapies, especially among those lacking sufficient health insurance who are often at highest risk of CVD and other DM-related outcomes. Automated systems aimed to improve use of these and other evidence-based therapies in appropriate patient populations are crucial to optimize quality of healthcare.

## Data Availability

Qualified researchers may request access to data from the *All of Us* study. Complete data are available at: allofus.nih.gov.
